# Directed evolution of enzymes at the crossroads of tradition and innovation

**DOI:** 10.1002/2211-5463.70271

**Published:** 2026-05-12

**Authors:** Maria Tomkova, Andrej Mirossay, Erik Sedlak

**Affiliations:** ^1^ Center for Interdisciplinary Biosciences, P. J. Šafárik University in Košice Košice Slovakia; ^2^ Department of Pharmacology Faculty of Medicine, P. J. Šafárik University in Košice Košice Slovakia; ^3^ Department of Biochemistry Faculty of Science, P. J. Šafárik University in Košice Košice Slovakia

**Keywords:** biocatalysts, directed evolution, protein engineering, screening, selection

## Abstract

Directed evolution has become a central methodology for engineering proteins with improved or entirely new functions, enabling applications across biotechnology, medicine, and synthetic chemistry. By iteratively coupling genetic diversification with screening or selection, directed evolution allows functional optimization even when detailed structural or mechanistic knowledge is unavailable. While display‐based selection platforms have enabled the efficient evolution of binders from extremely large libraries, enzyme evolution relies primarily on quantitative screening strategies that preserve genotype–phenotype linkage, often through compartmentalization. This review focuses primarily on enzyme directed evolution, using binder evolution as a comparative reference point to highlight key methodological differences and parallel advances. Major technological advances—including *in vitro* emulsions, droplet microfluidics, ultrahigh‐throughput sorting, genetically encoded biosensors, and alternative detection modalities—have dramatically expanded screening capacity and analytical resolution. We also discuss why stability remains a central constraint on evolvability, why assay design continues to limit translational relevance, and how failures such as surrogate‐substrate bias, droplet leakage, tracking errors, and overfitted machine‐learning models can misdirect campaigns. By integrating classical strategies with emerging continuous and data‐driven approaches, enzyme directed evolution is moving toward more predictive, automated, and industrially translatable workflows.

## Graphical abstract

An iterative cycle of data‐driven enzyme optimization comprising four stages: genetic diversification of a template enzyme, expression of protein variants, high‐throughput evaluation, and machine‐learning‐guided redesign of the next variant library. This closed‐loop framework reflects the trajectory of modern directed evolution toward predictive, automated, and industrially translatable workflows. Created in BioRender. Tomkova, M. (2026) https://BioRender.com/p4g9d79.

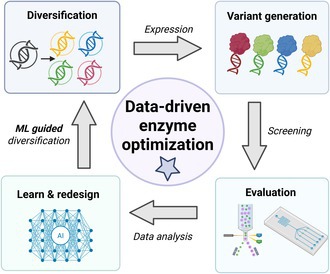

**Abbreviations**
AADS: absorbance‐activated droplet sorting
CSR: compartmentalized self‐replication
DMS: deep mutational scanning
FACS: fluorescence‐activated cell sorting
HTS: high‐throughput screening
IVC: *in vitro* compartmentalization
MADS: mass‐activated droplet sorting
ML: machine learning
OrthoRep: orthogonal DNA replication system
PACE: phage‐assisted continuous evolution
w/o: water‐in‐oil
w/o/w: water‐in‐oil‐in‐water

The first concept of *in vitro* Darwinian evolution was demonstrated as early as the 1960s, when Spiegelman and colleagues first observed self‐duplicating RNA molecules evolving in a cell‐free system [1]. Further development in this field was significantly accelerated in the mid‐1980s by the introduction of the polymerase chain reaction (PCR), which enabled the rapid amplification of genetic material [2, 3] **(**Fig. 1 **)**. As directed evolution relies fundamentally on DNA diversification, major progress came from the development of error‐prone PCR [4] and DNA shuffling [5]. These techniques made it possible to create large and diverse libraries of protein variants. Today, DNA libraries can be generated using approaches ranging from random mutagenesis and recombination to targeted methods, including CRISPR‐ and retroelement‐based mutagenesis, as well as *in vivo* diversification [6, 7, 8]. Moreover, the integration of computational tools and machine learning (ML) has enabled the design of ‘smart’ libraries, which significantly enhance the efficiency of the evolutionary process by focusing on the most promising regions of the protein sequence [9, 10]. DNA libraries are then used to produce a vast array of candidates with different sequence changes, which are subsequently subjected to screening or selection to identify variants with improved or new traits. The repetition of such diversification‐evaluation process forms the core of directed evolution [11, 12, 13]. Over the past 25 years, these techniques have evolved significantly, allowing us to modify proteins, improve enzyme activity, or create new catalysts for non‐natural reactions used in academia and industry [6, 7, 8]. This progress was recognized by the 2018 Nobel Prize in Chemistry, awarded to Frances H. Arnold for the directed evolution of enzymes, and to George P. Smith and Sir Gregory P. Winter for the phage display of peptides and antibodies [14].

**Fig. 1:**
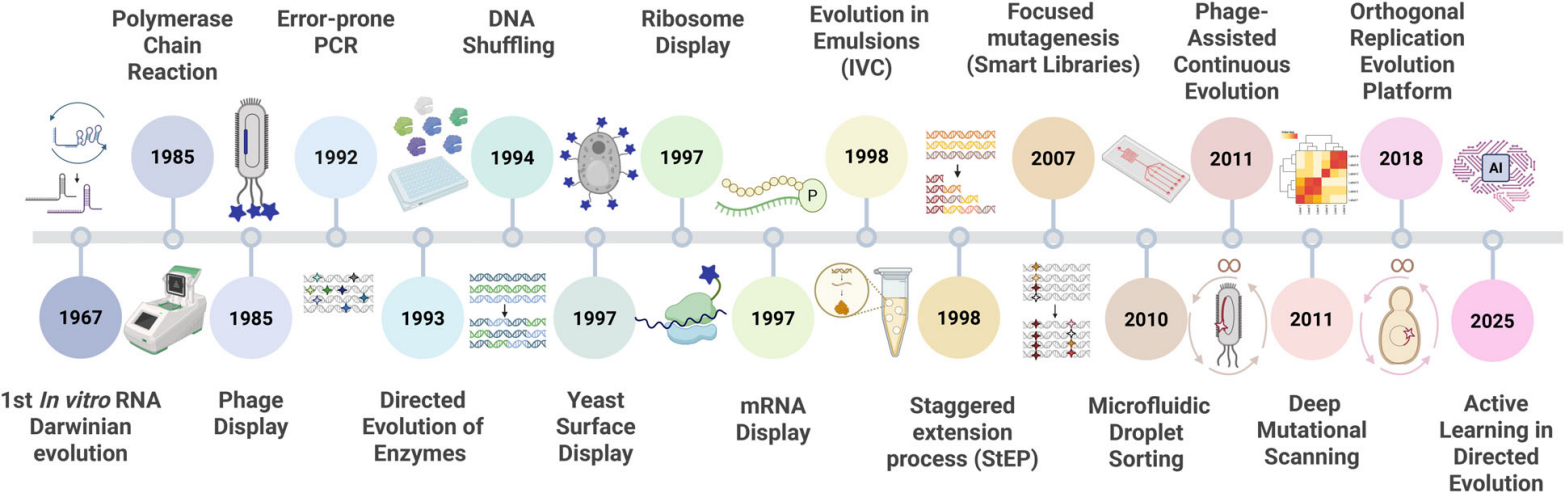
Timeline of milestones in directed evolution. Key advances from the first *in vitro* RNA Darwinian evolution [1], PCR and phage display [2, 15], early directed evolution and mutagenesis strategies [4, 5, 11], display and compartmentalization technologies [12, 13, 16, 17], to smart libraries, high‐throughput screening, continuous evolution, deep mutational scanning, and machine‐learning‐assisted directed evolution [18, 19, 20, 21, 22, 23, 60]. Created in BioRender. Tomkova, M. (2026) https://BioRender.com/zg5z0a9.

Based on these developments, directed evolution is now widely used as a laboratory approach to mimic and accelerate natural evolution. Unlike rational design, directed evolution can be applied even when little is known about the structure or catalytic mechanism of the target protein [24]. The core of directed evolution lies in the iterative cycle of diversification and evaluation, with the selection pressure being incrementally raised in each round **(**Fig. 2). Through this progressive repetition, the library becomes enriched with successful variants that withstand the increasing stringency, eventually driving the protein toward a new fitness peak [25]. A critical requirement of this process is the maintenance of genotype–phenotype linkage, defined as the physical or spatial coupling between a genetic variant and the functional properties of the protein it encodes. In addition, the process follows the fundamental rule that ‘you get what you screen for’ [24]. Therefore, the design of a robust discovery strategy is crucial, as the evolutionary path is driven by the specific criteria used to prioritize and enrich better‐performing variants [26, 27].

**Fig. 2:**
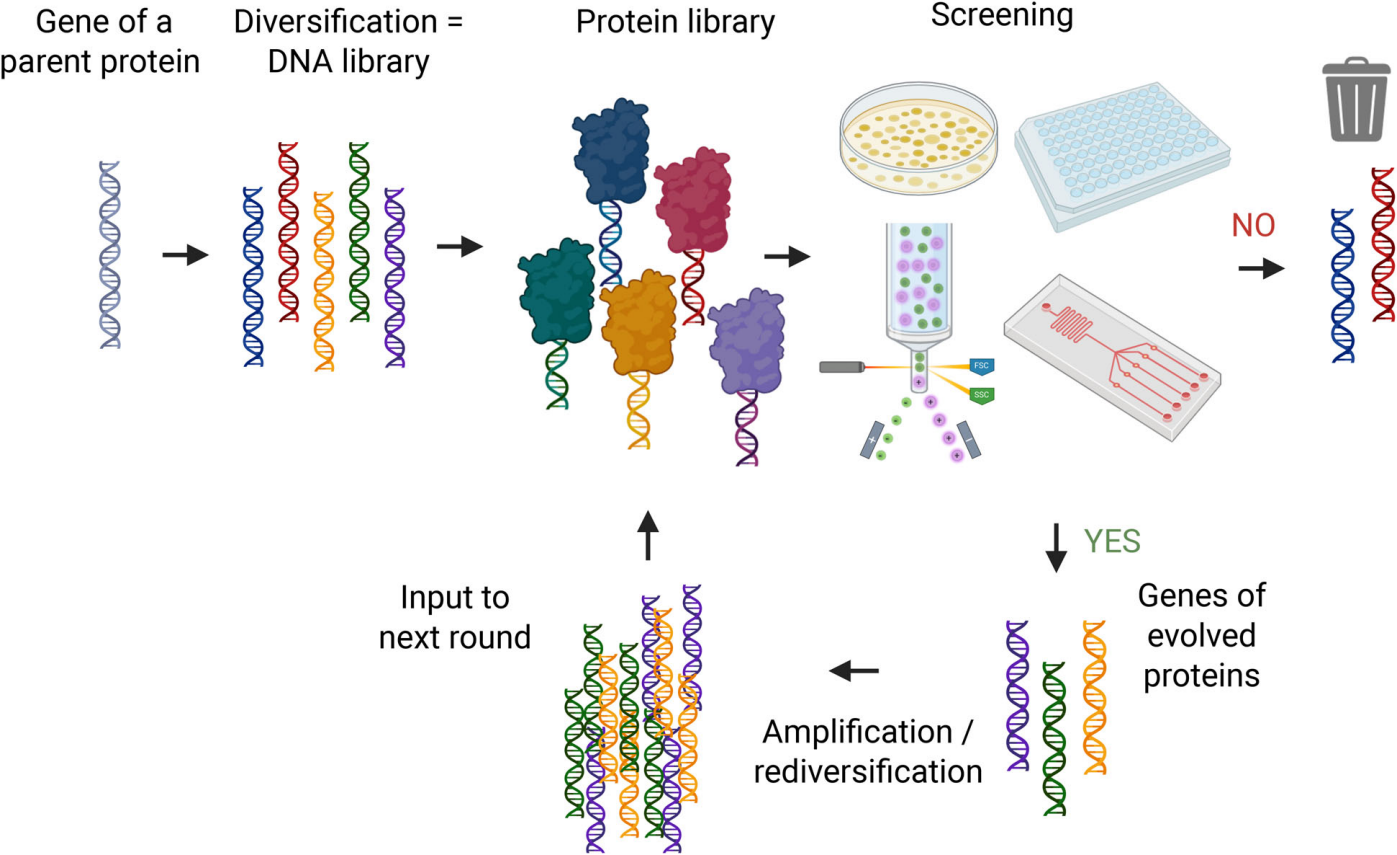
Directed evolution workflow for enzyme engineering. Schematic overview of a directed evolution cycle, illustrating library diversification, different screening methods, amplification, and iterative enrichment of evolved protein variants. Created in BioRender. Tomkova, M. (2026) https://BioRender.com/i1y7bc9.

## Binder *versus* enzyme evolution

Methods of directed evolution can be applied to a broad spectrum of proteins, ranging from binding molecules such as antibodies and peptides [28] to catalytic enzymes used in medicine, industry, and synthetic biology [8, 29]. Although these applications share the same core principles, strategies for evolving binders and enzymes often differ because they use distinct approaches to identify the best‐performing variants. Consequently, these methodologies have evolved along parallel yet partially distinct ways: One focused on display‐based evolution of binders [30], and the other often centered on screening‐driven evolution of enzymes [8, 26].

Display‐based evolution of binders relies primarily on selection, where a diverse population of variants is subjected to a defined selective pressure and variants that satisfy the binding criterion are retained. The outcome is therefore mainly enrichment across rounds, and it does not inherently provide a quantitative ranking of individual binders. Such selection mechanisms underpin phage, ribosome, or mRNA display, all of which maintain genotype–phenotype linkage through physical association rather than direct functional measurement [13, 15, 16, 30]. These technologies are now well‐established. For more than 30 years, they have been widely used in both academia and industry for antibody discovery, ligand identification, and mapping protein–protein interactions [30, 31, 32, 33, 34]. Their success mainly stems from the fact that binding affinity (i.e., dissociation constant) and epitope specificity are experimentally measurable properties inherent to the display format. Moreover, *in vitro* display techniques benefit from extraordinarily large library sizes [35, 36], which accelerate evolutionary exploration.

Yeast surface display has further expanded the binder evolution toolkit by enabling FACS‐based screening of libraries containing up to 10^9^ variants, with precise control over selection stringency through antigen concentration gradients [12, 37]. Affinity maturation by yeast display allows iterative enrichment of high‐affinity clones, achieving sub‐nanomolar dissociation constants across antibody and nonantibody scaffold formats [30, 34]. Related developments include RAPiD/mRNA‐display‐derived macrocycle discovery platforms and next‐generation sequencing (NGS)‐informed selection strategies have provided a quantitative, population‐level view of library enrichment, enabling analysis of thousands of variants simultaneously and revealing epistatic interactions across sequence space [21, 38]. Rapid affinity maturation protocols that combine high‐stringency selection with deep sequencing further accelerate the discovery of optimized binders with defined specificity profiles. These advances in binder evolution offer important methodological parallels and contrasts to the screening‐driven approaches that dominate enzyme evolution and are discussed comparatively throughout this review (Table 1).

**Table 1:** Key differences between binder and enzyme directed evolution. The table compares fundamental parameters. Where appropriate, nuances and exceptions are noted to reflect the practical complexity of each approach. While binder evolution relies on high‐capacity selection systems, enzyme evolution requires quantitative screening or compartmentalization to accurately measure catalytic properties.

Parameter	Binder evolution	Enzyme evolution
Fitness metric	Binding affinity and specificity	Catalytic activity, selectivity, enantioselectivity
Evolved quality	Direct (binding affinity and specificity)	Indirect (surrogate substrate, two‐step approach)
Readout type	Predominantly binding/no‐binding (often binary); can be made semi‐quantitative via competitive elution or NGS‐based enrichment	Quantitative or semi‐quantitative activity measurement can be effectively binary when dynamic range is limited
Dominant strategy	Primarily selection‐based (variant pool)	Primarily screening‐based (individual variants)
Typical methods	Phage display, ribosome display, mRNA display, yeast surface display, plasmid display	Microtiter‐plate screening, droplet microfluidics, cell‐based biosensors
Library size	Very large (10^9^–10^13^ variants)	Smaller (10^4^–10^8^ variants)
Genotype–phenotype linkage	Physical linkage (phage, cell, ribosome, mRNA)	Compartmentalization (emulsion droplets, cell expression), spatial separation (microtiter plates, colonies), yeast display
Quantitative ranking	Limited for individual clones; NGS‐based enrichment can provide relative fitness scores across the library	Direct and quantitative (individual clone analysis)

**Fig. 3:**
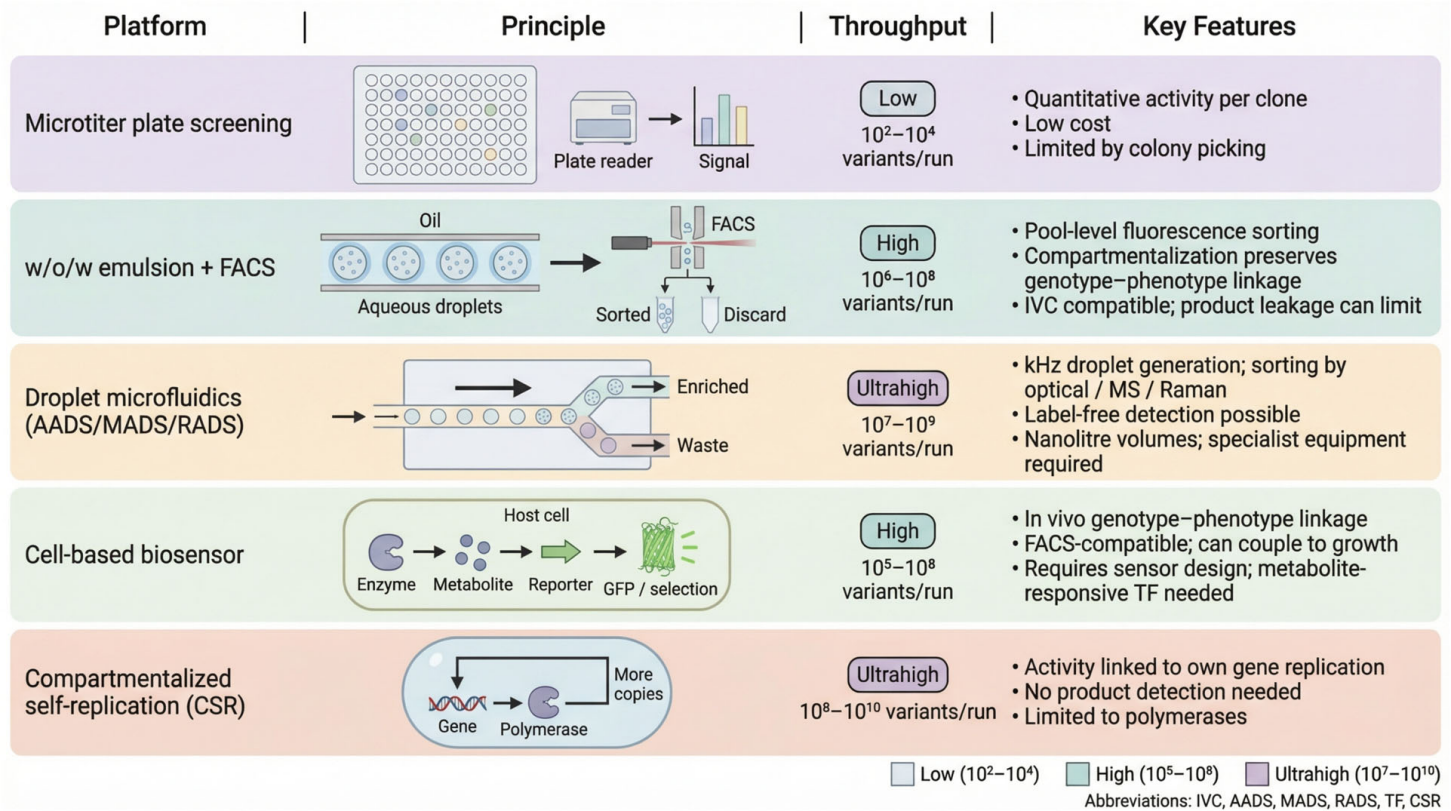
Overview of screening platforms in enzyme directed evolution. Comparison of commonly used screening platforms differing in throughput and assay format. Microtiter‐plate screening provides quantitative measurements at low throughput. Water‐in‐oil‐in‐water emulsions (w/o/w) combined with fluorescence‐activated cell sorting (FACS) enable high‐throughput sorting while maintaining genotype–phenotype linkage. Droplet microfluidics approaches, including absorbance‐activated droplet sorting (AADS), mass‐activated droplet sorting (MADS), and Raman‐activated droplet sorting (RADS). Cell‐based biosensors use intracellular reporters such as green fluorescent protein (GFP) to link metabolite production to signal output. Compartmentalized self‐replication (CSR) couples enzymatic activity directly to gene amplification, eliminating the need for external detection. Created in BioRender. Tomkova, M. (2026) https://BioRender.com/ftyhsmm.

In contrast, screening‐based approaches follow a different logic. They evaluate variants individually and generate quantitative or semi‐quantitative (rank‐order) measures of performance. These methods are particularly important for enzyme evolution, where the catalytic activity of each variant is generally assessed directly. Because enzyme reaction products typically diffuse, thereby breaking the genotype–phenotype linkage that directed evolution systems rely on, these methods often require compartmentalization or a mechanism that retains reaction products in close proximity to the variant that generated them [8, 17, 19]. As a result, platforms for enzyme directed evolution employ screening‐based approaches, such as microtiter‐plate assays, cell‐based biosensors, and compartmentalization strategies, including *in vitro* emulsion and, more recently, droplet microfluidics [8, 19] (Fig. 3). In contrast to the evolution of efficient protein binders, which primarily aims at decreasing the dissociation constant for a given ligand‐protein pair, enzyme evolution is inherently more complex, as it is not straightforward to simultaneously optimize both catalytic turnover and substrate affinity. Consequently, improvements in catalytic efficiency are often achieved in consecutive or staged optimization steps.

Together, these advances form the methodological basis of modern directed evolution. As outlined above, classical display technologies are generally not well suited for enzyme evolution, making enzyme engineering more challenging. The following sections outline the principal approaches and practical considerations used in directed evolution, *with emphasis on enzyme evolution*, using binder evolution as a comparative framework to highlight key mechanistic and methodological distinctions [8, 26, 30] (Table 1).

## Early stages of enzyme directed evolution

The first demonstration of enzyme directed evolution appeared in 1993, when Chen and Arnold evolved subtilisin E for activity in a non‐natural environment using sequential random mutagenesis and microtiter‐plate screening [11]. Although they evaluated only a few thousand protein variants (~ 4000), this study provided crucial proof of principle that enzymes could be evolved *in vitro*. The authors observed that beneficial mutations improving catalytic activity predominantly occurred on the protein surface, specifically within variable loop regions, rather than in the conserved structural core or directly within the catalytic site. Furthermore, the discovered mutational effects were nonadditive, providing early evidence for epistatic interactions.

Early enzyme directed evolution experiments typically relied on either qualitative agar plate‐based screening or microtiter‐plate‐based assays or their combination [5, 11, 39, 40]. In the case of agar plates, the screening principle was based on bacterial colonies producing enzymes that reacted with the surrounding medium. This resulted in a visual change, such as a change in substrate color or the formation of a ‘halo’ zone, a clear circle around the colony, which indicated substrate degradation. The enzyme activity in microtiter‐plate‐based assays was usually measured quantitatively by a spectrophotometer (measuring absorbance or fluorescence) [40, 41, 42, 43]. The throughput of these methods was relatively low, with a screening library size typically limited to around 10^4^ per experiment. Until the mid‐2000s, these low‐throughput formats remained the standard for evolution of biocatalysts.

## Selection‐based strategies for biocatalysts

Because these early screening methods had limited throughput, researchers naturally tried to apply selection‐based strategies to enzyme evolution, as these methods were inherently high‐throughput and enabled the simultaneous evaluation of extremely large libraries [13, 15, 16, 30]. To preserve the genotype–phenotype linkage, these approaches employed specialized chemistries that physically tethered reaction products to the display scaffold [44, 45, 46]. This enabled so‐called ‘single‐turnover’ selection, in which catalytic activity was coupled to the genotype through physical attachment of the reaction product to the phage particle displaying the enzyme. However, despite their conceptual success, such strategies were applicable only to a narrow range of reaction chemistries.

Critically, while this single‐turnover selection prevented product diffusion, it had significant technical disadvantages: it did not reliably reflect catalytic efficiency and, in principle, offered very limited resolution between active variants. Selection‐based approaches can, however, be rendered more informative when combined with competitive elution strategies or NGS‐based enrichment tracking, both of which introduce a degree of quantitative discrimination into what would otherwise be a binary enrichment outcome. Nevertheless, these nuances did not overcome the fundamental limitations of applying display‐based selection to enzyme catalysis in the general case.

Consequently, although these display‐based selection strategies provided important proof‐of‐principle demonstrations, they did not achieve widespread implementation as standard methods because of their technical complexity. Instead, alternative approaches enabling high‐throughput screening emerged.

## The need for compartmentalization

Effective high‐throughput screening of biocatalysts requires a measurable signal that reflects enzyme activity and stays linked to the gene that encodes the enzyme. Fluorescence was already widely used in plate‐based assays. Fluorescence‐activated cell sorting (FACS) allowed rapid screening and sorting of large cell libraries based on their fluorescent signals. The main challenge was to keep the fluorescent signal and the genetic information together, which led to the development of *in vitro* compartmentalization (IVC). Introduced by Tawfik and Griffiths in 1998 [17], this method used water‐in‐oil emulsions to create tiny aqueous droplets. Each droplet, typically ranging from 2 to 6 μm in size, acted as a cell‐like compartment that contains a single gene and its expressed enzyme. By trapping the enzyme and its reaction products together, IVC prevented diffusion and maintained the connection between the gene and its activity.

Since its introduction, the IVC strategy has evolved from relatively low‐throughput binary selection to fully *in vitro*, high‐throughput quantitative screening platforms. The pioneering study by Tawfik & Griffiths [17] used indirect detection of enzymatic activity through DNA survival after restriction digestion in water‐in‐oil (w/o) emulsion compartments. This approach was later extended to fluorescence detection, where a fluorogenic substrate generates a fluorescent signal that remains confined within each w/o droplet, allowing fluorescent droplets to be manually collected by microscopy [47]. The next major advance was microbead display, in which each gene and its expressed protein are physically linked to streptavidin‐coated microbeads inside w/o droplets and detected using fluorescent ligands [47, 48]. This was followed by the introduction of water‐in‐oil‐in‐water (w/o/w) double emulsions (Fig. 3), which made these compartments compatible with FACS and enabled high‐throughput screening at rates of up to 40 000 events per second [49, 50]. In parallel, compartmentalized self‐replication (CSR) emerged, where active polymerase variants directly amplify their encoding genes within compartments [51]. This direct coupling of enzymatic activity with gene amplification creates a positive feedback loop in which only genes encoding highly efficient polymerase variants are selectively amplified.

Beyond classical fluorescence‐based assays, several emerging high‐throughput screening (HTS) platforms have expanded the analytical capabilities of enzyme evolution [8]. Other HTS platforms and detection methods include *in vivo* strategies that increasingly rely on genetically encoded biosensors, in which transcription factor‐, RNA‐ or DNA‐based circuits convert intracellular metabolite formation into fluorescent or growth‐linked signals, enabling rapid selection directly in living cells [52, 53]. *In vitro* microfluidic platforms have introduced alternative droplet‐sorting modalities beyond fluorescence, including absorbance‐activated droplet sorting (AADS), which enables colorimetric detection at hundreds to thousands of droplets per second [54], and mass‐activated droplet sorting (MADS), which couples droplet microfluidics with electrospray mass spectrometry to enable label‐free detection of reaction products [55]. Raman‐activated droplet sorting further allows chemically specific, nondestructive analysis without the need for reporter substrates [56, 57]. Collectively, these approaches overcome key limitations of fluorogenic assays, including the requirement for chemically modified substrates that may not fully recapitulate native substrate behavior and broaden the range of enzymatic reactions and substrates that can be accessed in HTS‐driven directed evolution [8].

## Ultrahigh‐throughput screening

A decisive breakthrough came in 2010, when Agresti et al. demonstrated the sorting of millions of droplet‐based enzymatic reactions per hour [19]. Using a microfluidic droplet‐generation platform, they produced highly uniform, picoliter‐scale aqueous droplets dispersed in an inert oil phase, with each droplet functioning as an isolated microreactor containing a yeast cell displaying an enzyme variant. This precise control over droplet size and composition enabled quantitative screening of enzyme libraries approaching up to 10^8^ variants. Although both classical IVC and droplet microfluidics rely on FACS, microfluidics differs by offering precise droplet generation, thereby enabling truly quantitative ultrahigh‐throughput screening. As a result, enzyme directed evolution reached throughput levels that had previously been attainable only in display‐based binder selections.

## Current trends in biocatalyst directed evolution

### Continuous evolution

**Fig. 4:**
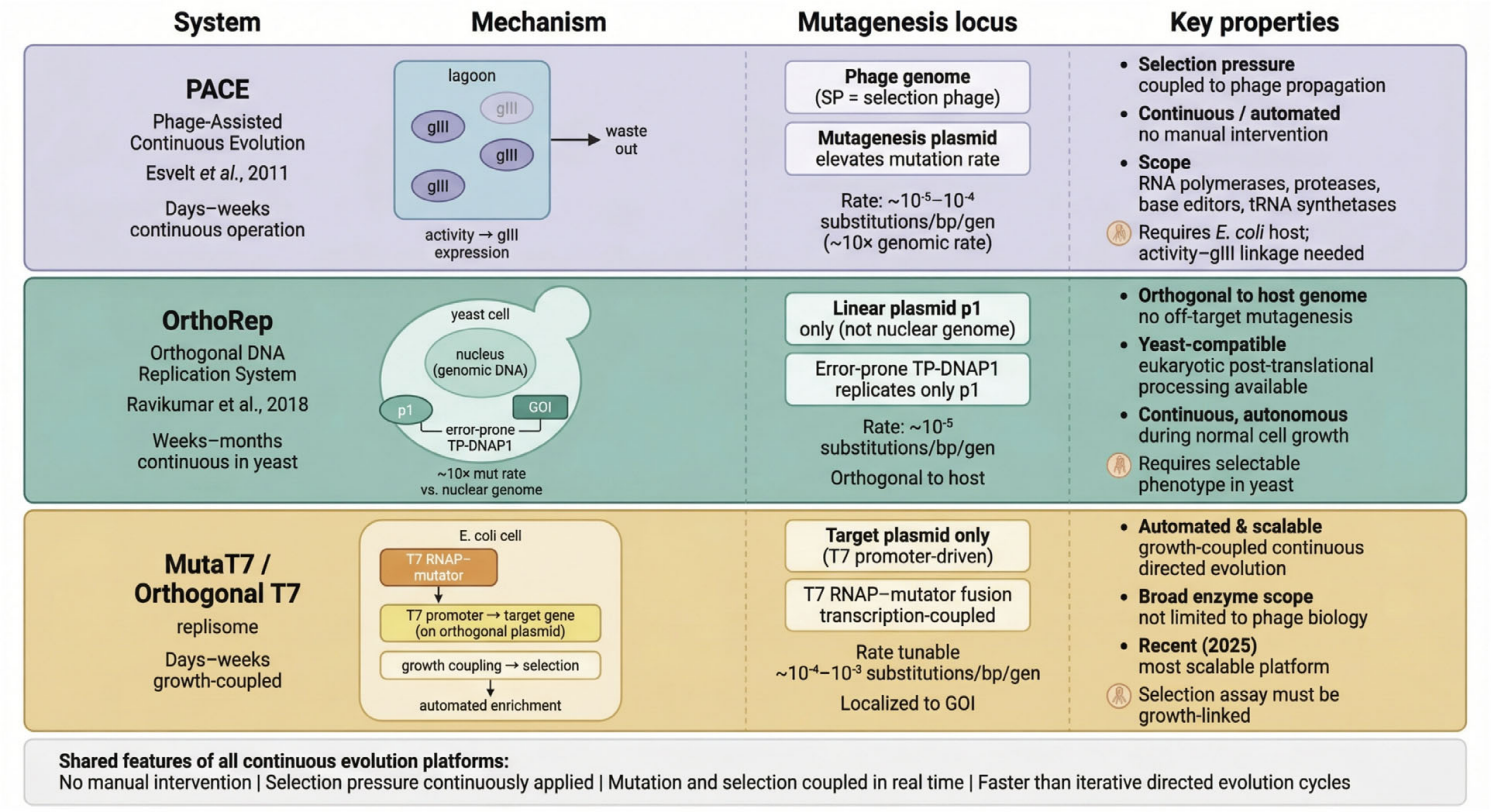
Overview of major continuous evolution systems for enzyme engineering. Phage‐assisted continuous evolution (PACE) links enzyme activity to phage propagation via gene III (gIII). OrthoRep (orthogonal DNA replication system) uses an error‐prone DNA polymerase to replicate a dedicated plasmid independently of the host genome. The MutaT7/orthogonal T7 system employs T7 RNA polymerase fused to a mutator enzyme for transcription‐coupled mutagenesis of target genes. All systems enable continuous mutation and selection without iterative manual cycles. Created in BioRender. Sedlák, E. (2026) https://BioRender.com/97p66sg.

Traditional directed evolution is limited by labor‐intensive repetition of cycles. Enzymes can now be evolved continuously inside living cells without iterative human intervention. These methods allow for autonomous, long‐term mutagenesis and selection without repeated manual cycles of classical directed evolution (Fig. 4). Among these, phage‐assisted continuous evolution (PACE) is the most widely adopted platform for continuous protein evolution, and it has been extensively applied to enzyme optimization in bacterial hosts [20, 58, 59]. PACE achieves continuous selection by linking the desired activity to the production of infectious progeny phage, enabling evolutionary rounds on an hour timescale. Using the PACE system, novel T7 RNA polymerase variants were evolved that recognize distinct promoters, initiate transcription with ATP instead of GTP, or initiate transcription with CTP. In total, approximately 200 rounds of evolution were completed within an eight‐day period.

An orthogonal DNA replication system (OrthoRep) with mutation rates several orders of magnitude above natural levels [22] enables continuous evolution of the gene of interest in yeast. OrthoRep achieves very high target gene mutation rates without affecting the host genome and allows evolution in many independent replicates, making it the most established continuous evolution system for eukaryotic hosts. A recent advance, the orthogonal T7 replisome system, introduces a fully orthogonal DNA replication apparatus in *E. coli* that supports continuous hypermutation of target genes at rates far exceeding genomic error thresholds, while preserving host genome integrity [60]. This platform expands the toolkit for bacterial continuous evolution and enables rapid traversal of protein fitness landscapes with minimal perturbation to the host cell. Additional approaches include EvolvR, a CRISPR‐based continuous evolution technique that enables precise, locus‐specific mutagenesis of selected gene regions [61], and MutaT7, which couples T7 RNA polymerase with a cytidine deaminase to achieve transcription‐coupled mutagenesis in a growth‐linked manner [62]. Collectively, continuous evolution platforms reduce cycle times and substantially accelerate laboratory evolution.

## *In silico* (machine learning) directed evolution

Deep mutational scanning (DMS) provides an unprecedented experimental map of how sequence variation affects protein function by quantifying the effects of thousands to millions of substitutions in parallel [21, 38]. These high‐resolution sequence–function landscapes reveal key residues, epistatic relationships, and mutational tolerance, creating rich datasets that fuel computational modeling and predictive design. Machine learning harnesses these data to infer complex, nonlinear relationships between sequence and function, enabling prediction of variant fitness and prioritization of beneficial mutations far more efficiently than unguided screening [9, 10, 63].

Active‐learning and iterative model‐guided optimization frameworks, in which ML models suggest new variants based on accumulated experimental results, have been shown to reduce experimental burden and accelerate the discovery of improved enzymes [23, 64]. Recent advances further demonstrate that combining ML with biophysical and structural information, for example via protein language models, physics‐based descriptors, or differential learning strategies, can improve prediction accuracy and enable extrapolation beyond locally sampled sequence neighborhoods [65, 66]. Protein language models trained on evolutionary sequence data have enabled zero‐shot prediction of mutational effects on stability and function at proteome scale, substantially reducing the need for large labeled experimental training sets [67]. The integration of autonomous experimental platforms with ML decision‐making has further enabled closed‐loop enzyme optimization campaigns, in which computational predictions and experimental feedback are coupled in real time to accelerate convergence on improved variants **(**Table 2 **)** [23].

**Table 2:** Machine‐learning‐guided directed evolution: principal methods and applications.

Method/model type	Data type	Application	Representative example
Deep mutational scanning (DMS) + regression/classification	Single‐mutation fitness scores (high‐throughput experimental)	Fitness landscape mapping; prediction of variant activity and stability	Stability–function prediction for enzyme families [21, 71]
Active learning / Bayesian optimization	Small iterative experimental datasets	Enzyme optimization with minimal screening burden	Active‐learning‐assisted directed evolution reducing experimental rounds ~10‐fold [23]
Protein language models (PLMs; e.g., ESM)	Evolutionary sequence data (unsupervised pretraining)	Zero‐shot fitness prediction; stability scoring; library prioritization	ΔΔG prediction at proteome scale [67]
Ensemble ML (random forest, neural networks)	Combinatorial variant libraries	Multi‐site combinatorial library design; prediction across sequence neighborhoods	ML‐assisted combinatorial protein evolution [63, 65]
Closed‐loop / autonomous platforms	Iterative HTS data with robotic feedback	Autonomous enzyme engineering without manual design cycles	Cell‐free ML‐guided enzyme optimization [23]

Despite this progress, predictive performance remains sensitive to data quality, dataset bias, and limited interpretability, underscoring the need for standardized experimental datasets and rigorous validation. Nevertheless, as high‐throughput experimental methods continue to generate richer training data and computational models become more sophisticated, ML‐guided strategies are poised to become central components of directed evolution, enabling more efficient navigation of protein fitness landscapes and faster discovery of optimized and novel biocatalysts.

## Stability in enzyme evolution: Challenges and solutions

Protein stability is a central and often limiting factor in directed evolution, for reasons rooted in the fundamental biophysics of protein structure. Natural proteins exist only marginally above their unfolding free‐energy threshold under physiological conditions ‐ a consequence of evolutionary pressures that have minimized the energetic cost of protein synthesis rather than maximizing structural robustness [68, 69]. This marginal stability severely restricts mutational tolerance: because most mutations are destabilizing, the introduction of beneficial functional mutations frequently comes at the cost of reduced thermodynamic or kinetic stability, creating a fundamental trade‐off between evolvability and folded‐state integrity [68, 70]. Large meta‐analyses of deep mutational scanning data confirm that predicted destabilization strongly correlates with loss of function across diverse protein families [71]. As a consequence, maintaining or recovering stability during directed evolution is a prerequisite for sustained evolutionary progress: variants that unfold or aggregate under selection conditions cannot be functionally evaluated, effectively shrinking the accessible sequence space and biasing the population toward structural, rather than functional, improvements.

Addressing this stability barrier has driven the development of a range of complementary strategies. Miniaturized experimental platforms enable parallel measurement of melting temperatures for hundreds of variants simultaneously, allowing stability to be incorporated as an early filter in directed evolution workflows [72]. Thermodynamic characterization by differential scanning fluorimetry and nanoscale calorimetry has been adapted for high‐throughput formats compatible with microfluidic and robotic pipelines. At the same time, protein language model‐based approaches can predict ΔΔG values almost instantly, making proteome‐scale *in silico* saturation mutagenesis feasible [67]. These computational stability predictors enable rapid prescreening of large libraries to exclude highly destabilized variants before experimental evaluation, substantially reducing wasted screening effort. AI‐based protein stability predictors have therefore become an increasingly important component of integrated directed evolution workflows, enabling early identification and exclusion of unstable variants before costly experimental screening. Despite this progress, current AI‐based stability predictors are still limited by dataset bias, poor generalization across diverse protein families, and low interpretability, highlighting the need for more diverse experimental data, especially for stabilized and hyperstable variants [73]. Recent reviews on PETase engineering illustrate the growing role of interdisciplinary approaches and ML in addressing stability challenges in industrial enzyme design [74].

## Evolution of new scaffold enzymes

Directed evolution allows researchers to move beyond the boundaries of natural biochemistry and design enzymes that catalyze reactions not found in nature, opening the door to genuinely new catalytic functions [25, 26, 75]. By reshaping existing protein scaffolds, often in combination with metal cofactors, enzymes have been engineered to perform carbene and nitrene transfer reactions, form carbon–carbon bonds, and selectively functionalize otherwise inert C‐H bonds [76, 77, 78]. Remarkably, many of these artificial enzymes achieve activities and selectivities that approach those of established chemical catalysts while operating under mild and environmentally benign conditions [79, 80, 81, 82].

**Fig. 5:**
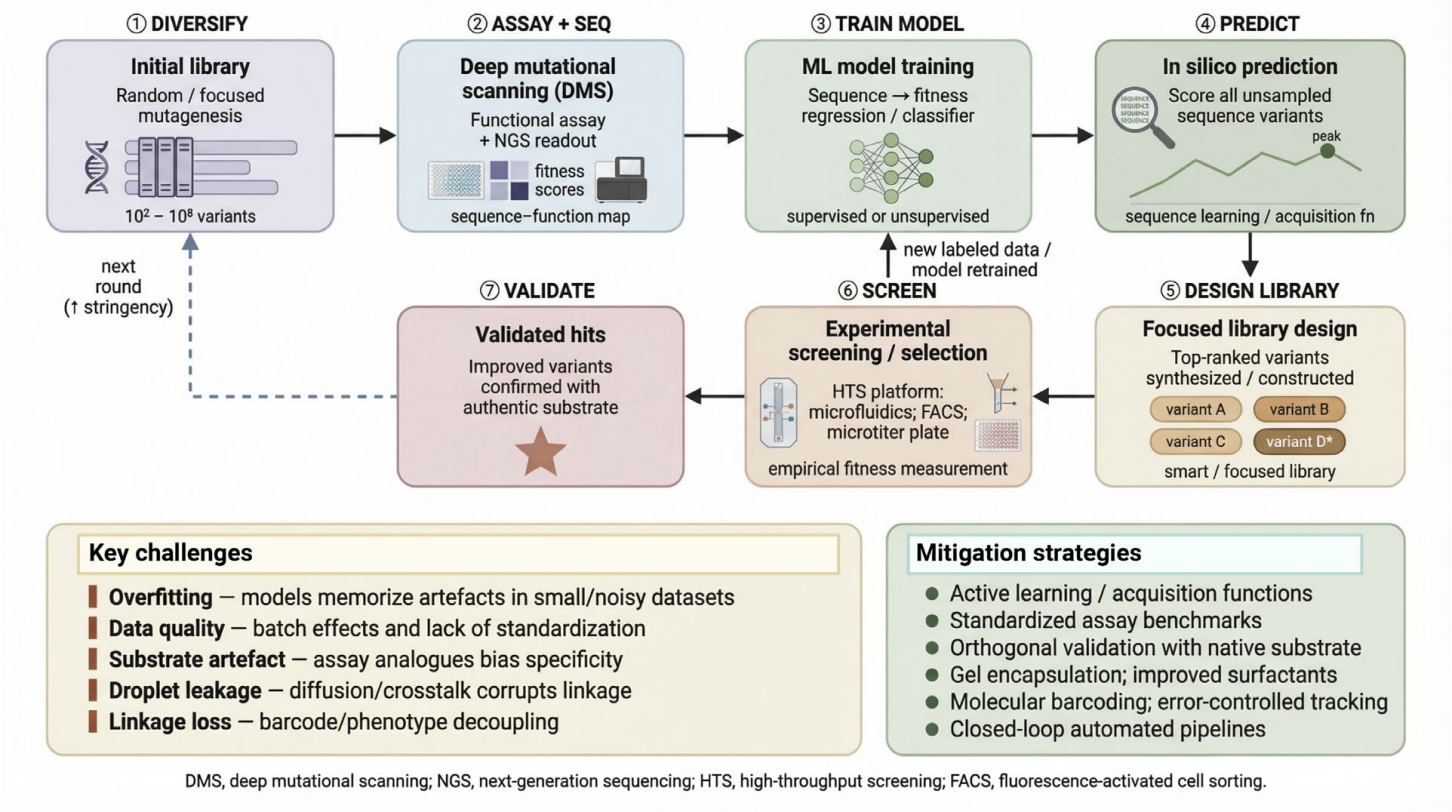
Modern integrated directed evolution pipeline. Schematic of a closed‐loop enzyme engineering workflow integrating library diversification [random, targeted, and machine‐learning (ML)‐guided], ultrahigh‐throughput droplet microfluidic screening, data analysis, and iterative ML model updating. The pipeline incorporates stability prefiltering and NGS‐based enrichment analysis at multiple stages to maximize the quality of each evolutionary round. Created in BioRender. Tomkova, M. (2026) https://BioRender.com/jp62gr5.

In most cases, these new reactions do not emerge fully optimized but originate from weak promiscuous activities or minimally active designed scaffolds, which are progressively refined through iterative rounds of mutation and selection [26, 77]. Directed evolution enables gradual reshaping of the active site, fine‐tuning of the metal environment, and stabilization of reactive intermediates that natural enzymes rarely accommodate [76, 77]. The process has been further accelerated by ultrahigh‐throughput screening technologies and ML‐guided library design, which help identify productive mutational pathways and focus experimental effort on the most promising regions of sequence space [8, 9, 10, 23] (Fig. 5). Together, these advances shorten evolutionary campaigns and make the discovery of efficient catalysts for new‐to‐nature reactions increasingly routine rather than exceptional. At the same time, achieving robust activity, scalability, and long‐term stability under industrial conditions remains a central challenge, underscoring the need for continued integration of protein engineering, advanced screening platforms, and data‐driven design strategies [8, 74].

## Challenges

Despite remarkable advances, high‐throughput enzyme evolution still faces several fundamental challenges that limit the translation of laboratory screening results into robust real‐world performance. A major bottleneck lies in the design of screening assays, which often rely on simplified surrogate substrates or artificial reaction conditions that poorly reflect industrial process parameters or physiological environments, leading to discrepancies between HTS performance and practical catalytic efficiency [68]. A well‐documented example is the use of fluorogenic substrate analogs, which can introduce substrate bias: because the fluorogenic group chemically alters the substrate, the evolved enzyme may acquire selectivity for the modified analog rather than the intended native substrate, a phenomenon sometimes termed fluorogenic substrate artifact [8]. Similarly, colorimetric or chromogenic surrogate substrates may select for altered promiscuous activities that do not translate to the target reaction, necessitating orthogonal validation assays with authentic substrates before selected variants can be considered genuinely improved.

In droplet microfluidic systems, product leakage between droplets represents a persistent technical challenge. Even with optimized fluorocarbon oil formulations and surfactants, slow passive diffusion or transient droplet coalescence events can allow fluorescent products or substrates to migrate between compartments, thereby corrupting the genotype–phenotype linkage that these platforms are designed to maintain [8]. This leakage effect is particularly acute for small, hydrophobic fluorogenic products and can lead to false enrichment of nonproductive variants during FACS‐based sorting. Strategies to mitigate leakage include the use of encapsulation in gel microparticles, enzymatic substrate injection immediately before sorting, and the development of surfactants with improved partitioning properties [83].

Another persistent challenge is the reliable maintenance of genotype–phenotype linkage when extremely large libraries are screened, particularly in pooled or droplet‐based formats. Loss of linkage, arising from droplet coalescence, cell lysis, or barcode misassignment during sequencing, can introduce systematic errors that confound the identification of genuinely improved variants. Sophisticated strategies such as molecular barcoding, compartmentalization, and error‐controlled tracking are increasingly employed to detect and correct such events but add experimental complexity and cost [8].

Data quality represents an additional limiting factor for ML‐guided evolution. ML models trained on small, noisy, or biased experimental datasets are prone to overfitting, in which the model memorizes training data artifacts rather than learning generalizable sequence–function relationships. Overfitted models may confidently predict high‐fitness variants in regions of sequence space that are poorly sampled or biologically implausible, leading to wasted experimental effort in subsequent screening rounds. This challenge is compounded by the fact that experimental datasets generated across different laboratories and platforms frequently lack standardization, suffer from noise and batch effects, and capture only a narrow subset of sequence–function relationships [74]. Moreover, the vast combinatorial size of protein sequence space, coupled with experimental biases introduced during library construction and screening, means that only a minute fraction of potentially beneficial variants can be experimentally explored.

Assay artifacts represent a final category of challenge that spans both screening and selection‐based platforms. These include autofluorescence of cell lysates that elevates background in fluorescence‐based assays, nonenzymatic background reactions that generate false positives in colorimetric assays, and growth advantages conferred by off‐target mutations that confound growth‐coupled selection systems. Rigorous controls, counter‐selection strategies, and orthogonal validation of top‐ranked variants are therefore essential components of any high‐throughput directed evolution campaign.

To address these limitations, the field is increasingly moving toward integrated and automated discovery pipelines that combine microfluidics, robotics, advanced analytical detection, and data‐driven computational design to improve throughput, reproducibility, and decision‐making efficiency [8]. Such closed‐loop platforms enable tighter coupling between experiment and computation, allowing rapid learning from experimental feedback and more rational navigation of protein fitness landscapes (Fig. 5). Continued progress will depend not only on further technical innovation in screening technologies but also on improved data standards, benchmarking practices, and interoperability between experimental and computational workflows, ultimately enabling more reliable, scalable, and predictive enzyme engineering.

## Conflict of interest

The authors declare no conflict of interest.

## Author contributions

MT contributed to the conceptualization, funding acquisition, writing – original draft preparation, writing – review and editing. AM contributed to the funding acquisition, writing – review and editing. ES contributed to the conceptualization, funding acquisition, writing – review and editing.
